# Keyword Index for Volume 90

**DOI:** 10.1038/sj.bjc.6601978

**Published:** 2004-06-08

^137^Cs-*γ* rays 48

[^18^F]FETA 2232

^18^F-fluorodeoxyglucose (FDG) positron emission tomography (PET) 620

2D gel electrophoresis 1955

2D-DIGE 173

2-methoxyoestradiol 932

2-methoxyoestradiol-*bis-*sulphamate 932

2-nitroimidazole 2232

^3^H-thymidine 48

3-hydroxy-3-methylglutaryl coenzyme A inhibitors 635

5-fluorouracil 348, 526, 1710, 1715

6-min walk test 366

6-thioguanine 1666

*α*-linolenic acid 787

*α*-MSH 1457

*α*-tocopheryl succinate 1644

Ab 1563

ABCA2 2411

acidic pH 1842

actinomycin D 1498

activation 283

active immunotherapy 2032

active specific immunotherapy 263

acute lymphoblastic and myelogeneous leukaemia 756

ADAM9 1052

adenocarcinoma 1787

ADEPT 2402

adhesion 1796

adjuvant anthracyclines 962

adjuvant treatment 1343

adolescents 613

adriamycin 1498

advanced breast cancer 31

advanced gastric cancer 1329

aetiology 2149

African-American 2171

age 497, 638

age–period–cohort models 1756

aggressive lymphoma 1151

AGUS 2194

ALA 1660

ALA derivatives 1660

alcohol 2225, 2390

aminolevulinic acid 805, 1660

analgesics 2288

anaphylaxis 304

anaplastic astrocytoma 1469

anastrozole 590, 1733

androgen 153

androstenedione 153

angiogenesis 206, 561, 1429, 1464, 1620, 2053

angiogenesis factors 245

angiogenesis inhibition 2418

angiogenesis inhibitors 1

angiotensinogen 1058

ANP 2073

anthracycline 917, 1740

antiangiogenic 430

antibody response 2402

antidepressants 314

anti-Fas 2025

anti-idiotypic antibody 2032

antioestrogen 20

antivascular 906

aplidine 2418

apoptosis 200, 270, 535, 561, 573, 705, 906, 944, 1058, 1285, 1644, 2017, 2025, 2370

area under the curve 353

aromatase inhibition 1733

aromatase inhibitor 20, 944

array-CGH 860

ASCUS 2194

Asian patients 1474

aspirin 93

association study 1989

astrocytoma 483

ataxia telangiectasia 866

*ATM* 522, 866, 1995

attitudes 106

AUC 2062

autologous melanoma vaccine 773

autophagy 1068

axillary lymph node 805

axillary lymph node dissection 1551

*AXIN1* 892

*β*-catenin 1100

BACs 860

Barrett's oesophagus 888

BB-3644 800

BC risk 2002

BCL-2 200, 236, 1933, 2025

B-CLL 2042

bethesda system 2194

betulinic acid 1672

bilateral testicular germ-cell tumours 55

bile acids 632

biliary tract carcinoma 1715

bioluminescence 1259

biomarkers 686

biopsies 383

bladder 542

bladder cancer 578, 1572, 2142

bladder cell lines 1679

blood flow 906

BMI 2138

BMPR1A 1230

BNP 2073

BNP 7787, 1654

body mass index 2176

bone metastases 1133

borderline epithelial ovarian neoplasias 1386

borderline tumours 1583

brachytherapy 2067

*BRCA1* 333, 1244, 1995

BRCA1 mutation 1492

BRCA1/2 1912, 2002

*BRCA2* 333, 483, 1244

BRCA2 mutation 1492

breast 423, 1780

Breast biopsy 595

breast cancer 20, 153, 160, 173, 182, 189, 236, 245, 393, 419, 476, 483, 582, 590, 652, 805, 911, 932, 968, 1040, 1120, 1133, 1138, 1211, 1216, 1349, 1361, 1414, 1422, 1429, 1479, 1486, 1531, 1551, 1612, 1740, 1912, 1920, 1926, 1942, 1989, 1995, 2118, 2123, 2131, 2135, 2149, 2153, 2225, 2344

breast cancer metastasis 253

breast cancer treatment 26

breast carcinoma 1538

breast MRI 1349

breast neoplasms 1343, 1378, 2138

breast radiography 383

bystander effect 1084, 1450

CA125 377

cachexia 996, 1274

CA-IX 985

caloric restriction 115

camptothecin 2261

cancer 60, 106, 408, 747, 752, 761, 882, 941, 1374, 1697, 1882

cancer pain 2288

cancer screening 383

cancer syndromes 860

cancer therapy 245

cancer treatment 328

cancer vaccine 1334, 1563, 2210

capecitabine 1190, 1312, 1329, 1740

carboplatin 87, 1318, 1498, 2062

carboxypeptidase G2 2402

carcinoid heart disease 2073

carcinoma of unknown primary site 1898

cardiovascular diseases 607

case–control 1784

case–control study 76, 146, 1792, 2145, 2176

CB 1954 1084

CC-5013 955

CC531 colorectal carcinoma 1259

CD105 1216

CD152 (CTLA-4) 2042

CD28 2042

CD9 expression 471

CDH1 874

*CDH13* 1029

Cdk4 503

*CDKN2A* (p16) 1594

cDNA microarray 2349

*CDX2* 701

cell culture 476

cell cycle 1583

cell cycle arrest 1672

cell malignancy 270

c-erb-B2 230, 2344

cervical adenocarcinoma 1784

cervical cancer 194, 1025, 1756, 1784, 1803, 2326

cervical intraepithelial neoplasia (CIN) 1025, 1407, 2194

cervical lesions 975

cervical neoplasia 146

cervical screening 1784

cervix neoplasms 1787

cetuximab 566

CGH 476, 492, 1422, 2411

*CHEK2* 888

chemoprevention 93, 736, 1011

chemoradiotherapy 348

chemoresistance 2370

chemotherapy 31, 65, 328, 353, 377, 573, 578, 601, 917, 968, 1259, 1306, 1498, 1502, 1704, 1885, 1898, 2112

Chernobyl 2219

*Chfr* 2013

childhood 139

childhood cancer 1016, 1771

children 613, 1882

China 1792, 2157

Chinese 1760

*CHK2* 888

*Chlamydia trachomatis* 1025

cholecystectomy 1753

cholelithiasis 1753

CHOP 372

chromatin 756, 761

Chromogranin A 2073

chromosome 10 1230

chromosome 17q21 2384

chromosome 3p 1983

chromosome deletion 860

CI-1033, ZD6474 2250

circulating DNA 1211

circulating tumour cells 443

cisplatin 87, 348, 359, 578, 968, 979, 1184, 1285, 1323, 1498, 1516

cisplatin resistance 1169

c-Jun/AP-1 2017

c-kit 1825

c-*kit* gene 2059

clinic correlations 1414

clinical benefit 1312

clinical efficacy 1733

clinical study 2317

clinical trials 1

clinical trials, phase I 2261

clustering analysis 216

c-Met 1555

c-*myc* 1612

Cockcroft and Gault 991

cohort 1386

cohort studies 1364, 1479, 2167

colon 882

colon adenoma 1955

colon cancer 1093, 1334, 1627, 1760, 1955, 1972

colony-forming assay 2356

colorectal adenoma 1591

colorectal cancer 76, 118, 306, 403, 483, 632, 712, 1003, 1029, 1190, 1230, 1429, 1479, 1502, 1620, 1666, 1707, 1710, 1753

colorectal carcinoma 1397

combination chemotherapy 87

combination phase I study 2092

combretastatin A-4 549

communication 106

comorbidity 2332

comparative genomic hybridisation 900, 1976

complementary alternative medicines 408

complications 2332

constipation 1397

continuous intravenous infusion 1156

core biopsy 595

cortisol 1129

cost 582

cost analysis 2067

cost-effectiveness 383, 1302

co-targeting 1825

counselling 2297

Cox regression 1176

COX-2 423, 701

COX-2 inhibition 712

CPT-11 1502

C-reactive protein 1707

creatinine clearance 991

cross-sectional study 787

CTL 1334

CTLs 1563

curative resection 1521

curcumin 1011

cutaneous malignant melanoma 497

cyclin D1 1235, 1933, 1942

cyclooxygenase-2 1760

CYFRA 21-1 2097

cystathionine beta-synthase 1969

cystatin C 1961

cytochrome P450 911

cytogenetic analysis 900

cytokines 955, 2312

cytomegalovirus 2149

cytotoxic RNases 270

cytotoxic T lymphocytes 1636

D2 gastrectomy 1727

DCIS 423, 1382

DDC 665

decision aids 333, 2123

definitive chemoradiation 70

deletions 503, 1983

demethylation 838

dendritic cells 1033, 1636

depression 787

deprivation 1367, 2142, 2153

desmoids 1443

dexamethasone 304

DFS 471

DHEAS 146, 153

diabietes mellitus 2171

diagnosis 393

diagnostic impact 1349

diarrhoea 1397, 2278

didemnin 2418

diet 128, 299, 632, 2167, 2278

differential display 1093

differentiation 728, 1833

disease response 1349

distress 2297

DNA damage repair 2203

DNA delivery 1252

DNA double-strand breaks 1297

DNA methylation 844

DNA repair 497

docetaxel 87, 348, 979, 1329

dose intense 601

dose intensity 353

doublets 87

Doxil® 1830

doxorubicin 1285, 2085

drug carriers 2261

drug delivery 917

drug delivery systems 2261

drug design 736

drug monitoring 343

drug resistance 573

ductal carcinoma *in situ* (DCIS) 1538

Dukes’ stage 1707

dyspnoea 366

early diagnosis 1780

early progression 2112

early-stage breast cancer 1543

EBAG9 2197

E-cadherin 1100, 1265

echinacea 408

economic analysis 397

education 638

efficacy 1156, 1190

EGFR 230, 1046

EGFR-targeting agents 566

eicosapentaenoic acid 996

EIF3S3 1040

EKB-569 2250

elderly 82, 1486

elderly and breast cancer 2332

embryonal tumours 1606

EMP/VBL vs EMP 100

endocrine 20

endocrine therapy 590

endometrial cancer 1756

endoscopic mucosal resection 672

endostatin 1627

endothelial cells 2418

endothelin-1 1577

endothelin-converting enzyme (ECE) 1577

endothelium 705

energy metabolism 996

England and Wales 1367

enteral/parenteral nutrition 2278

ephrin-B2 1620

epidemiologic studies 2171

epidemiology 139, 635, 1382, 1392, 2149, 2167

epidermal growth factor/receptor 449, 1563, 1679, 2250

*Epidermodysplasia verruciformis* HPV types 1777

epigenetic marking 756

epigenetics 515, 761

epinephrine 549

epirubicin 31, 962, 2131

Epstein–Barr virus 2149

ErbB-2 173

erbB-2/receptor 449

erbB-3 449

*ERCC2* 497

ERK 463

erlotinib 566, 2250

estrogen receptor 944

ethnicity 160, 1926

etoposide 1498

evidence-based medicine 26

exercise 366

expectations 582

experimental treatment 328

expression arrays 476

expression profile 216

fallopian tube carcinoma 1492

familial 1697

familial breast cancer 41, 321

familial risk 1765

family 1378

family and patient preferences 1474

famine 115

FAP 224

farnesyl protein transferase inhibitor 1508

Fas 1437

Fas Ligand 1437

fat 122

fat oxidation 1129

fatigue 2297

feeding 1129

FGFR4 genotype 189

FHIT 672, 2378

fibre 122

filter and nonfilter cigarettes 646

flat bone sarcoma 613

fluorescence redistribution 1450

fluoropyrimidines 526, 1190, 1740

fluorouracil 2338

FNA 595

foci 2356

foetal antigens 1374

folate 1969

folinic acid 1710

follicular adenoma 1600

follicular carcinoma 1600

follicular undifferentiation 492

follow-up 1144

fractional polynomials 794

free radicals 1274

fruits 2167

fulvestrant 236

fusion protein 1863

*γ*-catenin 1100

G : C → T : A mutations 1591

G1 phase 1672

G12C 1591

G388R mutation 189

gall bladder cancer 1516

gallstones 1753

GalNAc-T3 436

gap junction 1450

gastrectomy 1727

gastric adenocarcinoma 216

gastric adenoma 216

gastric cancer 206, 665, 672, 838, 1402, 1521, 1809, 1888, 1893, 2013

gastrointestinal stromal tumours 2059

GDEPT 1084

gefitinib 236, 566, 1679, 2250

gelatin zymography 1414

gemcitabine 31, 542, 1318, 1516, 1710, 1715, 2092

gender 2390

gene amplification 1612

gene expression 752, 1407, 1612

gene expression micro arrays 686

gene expression profiling 1814

gene polymorphism 526

gene profiling 1052

gene therapy 926, 1858

gene transcription 2006

genetic background 752

genetic counselling 321, 582

genetics 752, 1697, 1765

genomics 747

geographical variation 2153

germ cell cancer 1176

germ cell tumour 1169, 1526

germline mutation 503

GLC4-MITO 2411

glioblastoma 1058

glioblastoma multiforme 48, 1469

glioma 1068, 1469

glomerular filtration rate 991

GM-CSF 2025

goserelin 590

*GPC3* 1606

grade 1422, 1933

granulocyte colony-stimulating factor 1302

green tea 135, 1361

growth pattern 1429

GW-572016 2250

HAART 1526

hamartoma 701

haplotype 747

HBOC 1244

HCA661 1636

HCC 833

HDAC 535

head and neck cancer 1093, 1961, 1976

head and neck neoplasms 1323

health professionals 106

health services research 1882

heat shock protein 27 182

heat-shock protein 70 926

Heliox 366

hepatic epithelioid haemangio-endothelioma 1498

hepatitis B virus reactivation 1306

hepatoblastoma 1016

hepatocellular carcinoma 65, 1265, 1636, 2349

hepatocyte growth factor 822

hepatoma 1636, 2390

HER1 236

HER-2 423, 2344

HER-2/neu 443, 1814, 2032

herbal remedies 408

herceptin 1814

hereditary 1765

hereditary breast cancer 1244

hereditary cancer syndromes 510

herpesvirus-8 2145

heterozygosity 866

HGF 1555

high-dose chemotherapy 1169

histological grade 1538

histological type 646

histology 1138

histone acetylation 844

histones 761

HIV 1526

HIV Tat protein 1252

HMFGl 1863

hMre11 2356

HNPCC 882

Hodgkin's lymphoma 620, 522

Hoechst 33342 906

homing 1076

homocysteine 1969

hormonal contraceptives 1386

hormonal therapy 950, 2317

hormone independence 720

hormone replacement therapy 770

hormone resistance 1312

hormone-escaped prostate cancer 100

horseradish peroxidase 1858

hospital-based case–control study 646

hPGFS 1093

*hprt* 1666

HPV 1803, 2203

hTERT 1222

human 115, 1011, 1058

human foamy virus 2181

human kidney 2364

human papillomavirus (HPV) 638, 1025, 1407, 1777, 1949

human scribble 194

human T-cell lymphotropic virus type I (HTLV-I) 2181

hyperlipidaemia 635

hypermethylation 2013

hypermethylation of *MGMT* 874

hypersensitivity reaction 304

hypoxia 430, 728, 1235, 1429, 1842, 1858

hypoxia inducible factor1 1235

hysterectomy 1756

ibandronate 1133

ICIS 2256

Id-1 overexpression 1198

IDN 5390 1464

IFN-*γ* 2210

ifosfamide 1498, 2268

ifosfamide turnover 911

IGF-1 1076

IGF-1R 1825

IHC 471

IL-6 853, 2312

image analysis 430

imaging 2232, 2256

imatinib 2059

immunisation 48

immunogenicity 2402

immunohistochemistry 414, 672, 1052, 1222, 1555, 1572, 1933, 1955, 2197, 2338

immunomodulation 955

immunotherapy 985, 1033, 1334

*in utero* exposures 2225

*in vivo* 535

*in vivo* imaging 1259

*in vivo* systems 1464

incidence 55, 118, 1756, 1882, 2135

increased risk 41

induction chemoradiotherapy 979

inefficiency 752

infections 139

inflammation 1457

informatics 1115

information needs 1474

informational needs 1144

infusional 5-fluorouracil 1893, 2131

*INK4a*-ARF 503

inoperable 70

insulin 1129

integrin 1457

integrin *α*5*β*1 561

integrin *α*v*β*3 561

interactions 794

interferon 173, 794

interferon-*α* 626

interferon-*γ* 48

interleukin-2 626, 773, 1156

interleukin-6 419, 1129

interstitial pneumonitis 1526, 2080

interval cancer 393

intra-arterial chemotherapy 1323

intracellular localisation 194

intraperitoneal chemotherapy 403

invasion 253, 822, 1265, 1437, 1443, 1577

invasion and metastasis 455, 2349

invasive epithelial ovarian neoplasias 1386

invasiveness 712

involucrin 728

ionising radiation 866, 1297

Iran 1402

irinotecan 87, 306, 1502

irinotecan (CPT-11) 1508

isoforms 283

isolated limb perfusion 1830

JACC Study 135

Japan 646

JIP-1 2017

JNK 2017

karyotype 900

K-cadherin 1100

keratinocyte 1949

Ki-67 626, 1046, 1555

kidney 1654

KIT 2397

knockout mice 906

kola-nut 638

k-*ras* 1591

LAP protein 194

laxative 1397

leptin 1129

Leucocyte 2053

leucovorin 1715

leukaemia 139, 1771

life change events 1364

ligand 1620

light exposure 941

linkage analysis 510

lipid mobilizing factor (LMF) 1274

liver cancer 1636

liver metastasis 1429

liver transplantation 1498

liver tumour 1498

*LKB1* 701

LNCaP 2017

local recurrence 1920

locally advanced 968

locoregional recurrence 1543

LOH 1230

loss of heterozygosity 892, 1204, 2378, 2384

low-grade glioma 781

LSCC 1594

LSIL 975

luciferase 1259

lung adenocarcinoma 436

lung cancer, gene expression 1093

lung cancers 366, 397, 646, 787, 1125, 1222, 1555, 1972, 2288, 2378

lymph node dissection 1727

Lymphadenectomy 1888

lymphangiogenesis 693

lymphoma 620, 2145

LYVE-1 693

MACOP-B/VACOP-B 372

*MAGE* 838

magnetic resonance imaging 2326

magnetic resonance spectroscopy 781, 2326

major depressive disorder 310, 314

malignant germ cell tumours 601, 607

malignant melanoma 693, 770

malignant mesothelioma 1644

mammary gland 2225

mammography 393, 652

management 397

management LSIL 975

MAPK 283, 1046

markers 1933

Markov model 397

Markov modelling 1912

mass screening 2118

matrix metalloprotease inhibitor 800

matrix metalloproteinases 1414

maximum tolerated dose 2092

MBD3 1972

Mdm2 1285

MDR 1464

mean lethal dose 48

melanocortin 1457

melanoma 263, 1279, 1457, 1833

melanoma genetics 503

melatonin 941

mesna 1654

mesothelioma 1022, 1905

meta-analysis 93, 1302, 2097

metabolism 736

metalloproteinases 463

metastases 1531

metastasis 561, 693, 712, 752, 1230, 1279, 1437, 1842, 1877, 1976, 2053, 2186, 2344

metastatic breast cancer 36, 962

metastatic gastric cancer 1885

metastatic melanoma 773

metastatic process 443

metastatic renal carcinoma 794

metastatic renal cell carcinoma 1156

methionine synthase 1969

methotrexate 353

methylation 1606, 1972

methylation-specific PCR 1029

methylenetetrahydrofolate reductase 526

MGB2 686

MIB-1 626

MIBG 1842

MICA gene 1809

microarray 173, 752, 1120

micro-satellite instability 483, 2006

microsatellite stable 1666

microvessel density 206

migrants 2135

migration 230, 455

minichromosome maintenance proteins 1583

mismatch repair 2006

mithramycin A 2025

mitochondrial biogenesis 2390

mitomycin C 1893

mitosis 815

mitoxantrone 2411

*MLH1* 672, 1594, 2006

MMP expression 1443

MMPs 1679

model 2157

modifiers 752

molecular biology 833

molecular target therapy 2059

monoclonal antibody 985

morphological differentiations 720

mortality 607, 652, 1780, 2157

mortality trends 1022

motility 253

mouse models 752, 756

mPGES-1 701

*MSH2* 483, 2006

MSI 882, 1666

MSS 1666

MTA1 455

MTHFR 1969

MTT 48

MUC1 1863

MUC4 657

mucin phenotype 672

mucinous LMP 1204

mucins 657, 720

multicolour fluorescence *in situ* hybridisation 900

multidrug resistance 2411

multiple myeloma 1076

multiple partners 1374

multiple primary cancers 483

murine tumours 1842

mutation analysis 888, 1244

mutations 522, 866, 892, 1572, 1972

mutator phenotypes 1666

MYC 1040

MYH 1591

myofibroblast 822

n-6 polyunsaturated fatty acids 1760

nails 1392

NASBA 1407

N-cadherin 1100

necrosis 549

nedaplatin 1125, 2092

neoadjuvant 968, 1521

neoadjuvant chemotherapy 1349, 2326

neoadjuvant treatment 1323

neoplasm metastasis 705

neoplasms 1364, 2297

neoplastic progression 182

neovascularisation inhibitors 705

neuroblastoma 515, 2210

neutral endopeptidase (NEP) 1577

neutralising antibody 1068

night work 941

nitroreductase 1084

nm23 2186

NO signalling 2364

node-negative 1543

nongerm cell cancer 607

non-Hodgkin's lymphoma 1151, 1302

nonseminomatous germ-cell tumour 55

non-small-cell lung cancer (NSCLC) 82, 87, 359, 900, 979, 1184, 1704, 1905, 1983, 2092, 2097

nonsteroidal anti-inflammatory agents 93

Norway 1386

NOS 2364

NQO1 1989

NSCLC 1046

nuclear factor-*κ*B 1850

nuclear localisation 1833

nucleolar localization 1222

nurse 310

nutrition 118

nutritional status 2278

obesity 299

oesophageal cancer 70, 888, 892, 1402

oesophageal carcinoma 2067

oestradiol 146, 153, 590

oestrogen 153, 423, 770

oestrogen receptor 853, 1942, 2197, 2225

oestrogen suppression 1733

oestrone 146, 153

oestrone sulphate 146

old age 1151

omega-3 fatty acid 787

oncogenes 573, 1407

oral 1190, 1740

oral bisphosphonate 1133

oral cancer 1323

oral cavity cancer 2062

oral oestrogen 76

oral squamous cell carcinoma (OSCC) 2186

orthotopic environment 657

osteopontin mRNA 1796

osteosarcoma 613

outcome 2326

outcomes of consultants’ communication 321

ovarian cancer 476, 686, 810, 1318, 1627, 2167, 2197

ovarian carcinoma 678, 815, 874, 1204

ovarian neoplasms 377

ovary 1583

overexpression 1040, 2344

oxaliplatin 306, 1710

oxygen 366

oxygen regulation 728

p21 236

p38 kinase 463

p53 206, 1285, 1572, 2006, 2203

P70 S6 kinase protein 1543

paclitaxel 31, 36, 304, 359, 962, 968, 1125, 1169, 1318, 1740

paediatric 60

pain 1163

Pain Management Index 2288

palliation 2067

pancreas cancer 1198

pancreatic adenocarcinoma 657

pancreatic cancer 1052, 1129, 1893

pancreatic carcinoma 455

para-aortic radiotherapy 2305

paracetamol 1858

parental smoking 1016

parity 115, 1025

pathology 1382

patient care 1144

patient confidentiality 1115

patient preferences 1144

patient prognosis 1983

PCNSL 1969

PCR 1407

PCR-based assays 1777

PDT 1660

pegylated liposomal doxorubicin 1893

pelvic malignancy 2278

pelvic radiotherapy 2278

peptides 1334, 1563

performance status 1704

perfusion 430

perinatal 139

peripheral blood progenitor cell support 1318

peritoneal 1437

peritoneal dissemination 665

peritoneal metastases 403

PET 2232

pharmacodynamics 2268

pharmacogenetics 8, 526

pharmacogenomics 8

pharmacokinetics 343, 800, 932, 1011, 1508, 1654, 2085, 2261, 2268, 2317

phase I 800, 955, 1508, 2085, 2268

phase II 65, 100

phase II study 1898

photodynamic diagnosis 805

photodynamic therapy 1660, 1833

physical activity 299, 2138

physician–patient communication 328

PI3-kinase 853

pimonidazole 728

PKI-166 2250

plakoglobin 2384

plant foods 122

plasma 1654

plasmatic clearance 353

platelet-derived growth factor 1068

PLC-*γ*1 230

pleural cancer 1022

PLK1 414

PMLBCL 372

point mutation 1627

Polo-like kinase 815

poly-lysine 1252

polymers 2085, 2261

polymorphism 8, 419, 1809

poor prognosis 601

population mixing 1771

population pharmacokinetics 60

population-based 1382, 1572

porphobilinogen deaminase 1833

postmenopausal 76

postmenopausal breast cancer 122

potency 1733

potentiation 917

PPSC 1492

practice patterns 1885

predictive factors 1942

pregnancy 1374

premedication 304

premenopausal 1543

preoperative chemotherapy 1521, 2338

preserved food 1792

prevention 299

primary care 1479

primary central nervous system lymphoma 353

problem-solving therapy 310

prodrugs 1084

profiling 1120

progesterone 146

progestogens 770

prognosis 200, 419, 436, 678, 770, 1052, 1120, 1138, 1198, 1204, 1216, 1378, 1877, 1961, 2097, 2186, 2332

prognostic classifications 1176

prognostic factors 206, 613, 626

prognostic marker 189, 2349

prognostic score 1704, 1707

progression 752, 1422, 2053

projections 2157

proliferation 423, 463

proliferative activity 833

prophylactic salpingo-oophorectomy 1492

prospective cohort study 1361

prospective studies, 122, 128, 1397

prostate cancer 443, 510, 535, 720, 926, 950, 1040, 1163, 1392, 1577, 1792, 2176, 2312, 2317

prostate neoplams 2171

prostate-specific antigen (PSA) 1312

prostatic carcinoma 1312

prostatic neoplasms 93

prostatic neoplasms/receptor 449

prostein 1033

proteasome expression 1850

protein kinase B/Akt 2370

protein kinase C 1850

protein nitration 2364

Proteolysis-inducing factor 1850

provider delay 1479

PSK 1003

psychoeducational intervention 41

psychosocial screening 2297

PTEN 1230

quality control 1115

quality of life 996, 1163, 1312, 1747

quantitative real-time PCR 1531

quantitative traits 752

R115777 (tipifarnib) 1508

Rad50 2356

Rad51 2356

RAD51 gene 2002

radiation 542, 950

radiation therapy 578, 2062

radiation-induced gastrointestinal toxicity 2278

radical cystectomy 578

radiography 2118

radioimmunotherapy 1469

radiosensitivity 2356

radiotherapy 573, 1151, 1343, 1469

Raf 283

raloxifene 944

raltitrexed 1502

randomised controlled trial 1003

randomised trials 333, 950, 2112

*RAR-β* and *SYK* gene 874

rat 1830

rat mammary metastasis 1796

*RB1* 1594

real-time PCR 1211

real-time quantitative RT–PCR 1600

real-time RT–PCR 665

receptor tyrosine kinase 289

receptors 1620, 2344

RECIST 2256

recombinant adenovirus 1636

recombination 1297

rectum 882

recurrence 377, 436

recurrent ovarian cancer 2112

recursive partitioning 1176

registries 1364, 1378

relative survival 1367

relative survival rates 1138, 2153

renal 1235

renal cell carcinoma (RCC) 200, 515, 626, 985, 2364

renal function 60, 991

renin 1058

renin inhibitors 1058

repair 1297

replication 1297

response 377, 2256

resveratrol 736

RET 2219

retrovirus 2181

review 93

RhoC 2349

ribonuclease 1863

risk factors 1025, 1306, 1753, 1787

risks 408

S100A4 253

safety 1156

salts, 128

sarcoma 1830

sarcoma cell lines 1285

satisfaction 328, 582

scatter factor 822

SCC of head and neck 471

scFv 1863

screening 299, 314, 393, 1780, 1803

secondary malignancies 2305

secondary resistance 2059

second-line treatment 377

secretion 926

SEER 1771

selenium 1392

self-expanding metal stent 2067

seminoma 55, 1765

sensitivity and specificity 2118

sentinel lymph node biopsy 1551

sentinel lymph nodes 805, 1531, 1551

sequential chemotherapy 810

serial analysis of gene expression 1600

serous cystadenocarcinoma 1583

serous papillary ovarian cancer 1814

serous papillary uterine cancer 1814

serpin variants 833

serum 1211

serum creatinine 991

sex 2142

sex hormones 115

sexual habits 638

SHBG 146, 153

shortening 1747

signal transduction 1076

signalling 283

simian foamy virus 2181

single-nucleotide polymorphism 747, 2002

SIP1 1265

skeletal complications 1133

*SLIT2* 515

*SMAD4* 701

small cell lung cancer 1825, 1905

small-cell carcinoma 1720

smoking 118, 1787

SN 23862 1084

snail 1265

SNP^5^ 1989

socioeconomic inequalities 1367

sodium butyrate 535

sodium folinic acid 1893

solid tumours 2256

soluble guanylate cyclase 2364

somatic mutation 2390

South Asian 160, 1926

SP1049C 2085

specialist 1920

sphingolipid 917

squamous carcinoma 822

squamous cell carcinoma 1125, 1787, 1961

squamous cell carcinoma of the head and neck (SCCHN) 348

squamous-cell carcinoma of the conjunctiva 1777

stability 1863

stage 1486, 1704, 2142

stage I 2305

staging 620

statins 635

sterically stabilised liposomes 263

stilbene 736

stimulation 2042

stomach cancer 135

stomach neoplasms, 128

stress 106

stress; psychological 1364

stroma 822

stromal–epithelial interactions 1577

structural genetic abnormalities 900

subcutaneous administration 1156

subgroup analysis 794

substrate 283

sulindac 224

sulphatase 932

sulphatase inhibitor 932

supplements 408

suppressive subtractive hybridisation 216

surgery 1888, 1920

survival 652, 815, 1052, 1343, 1526, 1704, 1707, 1727, 1912, 1920, 1926, 2142

survival rate 1198

Sweden 1386

synergism 1644

synergistic antitumour effect 1672

synergy 1825

systematic review 1697

systemic inflammatory response 1704

T cells 1033

tamoxifen 590, 944, 1942

targeted intra-arterial chemotherapy 2062

TAT 230

taxanes 1464

taxotere 2131

T-cell activation 955

TCF 224

tegafur/uracil 306, 1003

telomerase 1222

telomerase activity 1983

temozolomide 781

teratoma 1765

terminally ill 1163

testicular cancer 1526, 1765

testicular germ cell tumours 2397

testicular seminoma 2305

testosterone 153

tetrasomy 1949

TGF-*β*1 822

TGF*β*2 657

thalidomide 955

the gastrointestinal tract 1720

thrombocytopenia 2062

thymidylate synthase 526

thymoma 2181

thyroid 414, 492, 1600

thyroid carcinoma 2219

thyroid cells and tumours 270

thyrosine kinase inhibitor 82

Tie-2/Tek 1216

time trends 652

TIMP-1 463

TIMPs 1679

tissue banking 1115

TNF 1279, 2025

TNF-*α* 2312

TNM stage 1138

tobacco 299

topoisomerase I inhibitor 343

topotecan 2268

total body irradiation 1526, 2080

toxicity 2305

TP 2338

*TP53* 1989, 1995

*TP53* mutation 678

TP53 protein accumulation 678

*TP53* tumour-suppressor gene 678

TPA 2017

TRAIL 1644

training 106

transcriptional coactivator 844

transcriptional regulation 1606

transdermal oestrogen 76

transfected cells 1796

transfection 2210

transgenic adenocarcinoma mouse prostate 926

transrepression 853

trastuzumab 36

treatment 310, 652, 1138, 1486, 2332

treatment decision making 2123

treatment delay 1343, 1926

treatment response 781

trefoil factor 3 1600

tributyrin 535

TRPS1 1040

tumorigenesis 1877, 1983

tumour 882, 1654, 2053

tumour angiogenesis 1198, 1627

tumour antigen 1033

tumour blood flow 549

tumour gene therapy 2210

tumour hypoxia 2232

tumour latency 2219

tumour marker 1877

tumour necrosis factor 853, 906

tumour necrosis factor-alpha 183

tumour vaccine 1636

tumour vasculature 430

tumour vessel 549

tumour-associated antigens 263

Turkmen 1402

tyrosine kinase 189, 1620, 2250

UFT 1003

Uganda 1777

ultrasoft X-rays 1450

uncoupling protein-2 1274

upregulation 2338

urinary 5-HIAA 2073

usual care 314

UV 2203

vaccine 2032

vegetables 2167

vegetarian 118

VEGF 206, 1076

VEGF-C 693

VEGF-R1 1216

VEGF-R2 1216

VHL 1235

vincristine 1498, 1672

vinorelbine 359

viscum fraxini-2 65

weekly paclitaxel 1184

weight 2138

weight loss 1129, 1905

Western blot 2356

Western Europe 1022

whole-pelvic radiation 950

Wilms’ tumour 515

Wnt signalling pathway 892

women 1386

written information 41

WX-G250 985

X chromosome 1606

*XPD* 497

yessotoxin 1100

